# Reward-reset interval timing drives patch foraging decisions through neural state transitions in dorsomedial striatum

**DOI:** 10.1101/2025.09.29.679309

**Published:** 2025-10-01

**Authors:** Elissa Sutlief, Shichen Zhang, Kate Foresberg, Rie Kaneko, Marshall G Hussain Shuler

**Affiliations:** Department of Neuroscience, Johns Hopkins University School of Medicine, 725 N. Wolfe Street, Baltimore, MD 21205, USA; Department of Neuroscience, Johns Hopkins University School of Medicine, 725 N. Wolfe Street, Baltimore, MD 21205, USA; Department of Neuroscience, Johns Hopkins University School of Medicine, 725 N. Wolfe Street, Baltimore, MD 21205, USA; Department of Neuroscience, Johns Hopkins University School of Medicine, 725 N. Wolfe Street, Baltimore, MD 21205, USA; Department of Neuroscience, Johns Hopkins University School of Medicine, 725 N. Wolfe Street, Baltimore, MD 21205, USA

## Abstract

Activities with diminishing returns pose a unique computational problem for the brain, requiring a combination of outcome evaluation and temporal tracking. Deciding the right time to stop one pursuit and move to alternatives is an important part of effective time management, yet it is unknown how the decision-making circuits of the brain determine the moment to switch. The dorsomedial striatum (DMS) mediates both goal-directed decision-making and interval timing—two functions that converge during patch foraging, where animals must time when to exit patches to maximize reward rates. We recorded extracellular activity from neurons in DMS while freely moving mice performed a patch-foraging task. Mice employed a ‘reward-reset’ strategy, primarily basing their exit decisions on the time since the last reward, but with the patch residence time and environmental reward-rate context also contributing to the intended time of departure. Individual neurons in DMS underwent discrete firing rate transitions at characteristic delays following each reward. These transition delays were distributed across the population, creating a cumulative signal that reached a threshold coinciding with patch exit. This population activity pattern spanned the intended reward-to-exit interval, compressing or expanding in accordance with patch residence time and changing environmental conditions. Fiber photometry recordings revealed phasic dopamine signals in DMS encoding reward prediction errors that reflected the declining reward probability over time. Our results provide insights into how DMS integrates its dual roles in timing and action selection to guide time investment strategies during foraging.

## 1. Introduction

Decision-making is usually thought of as the process of selecting from a menu of options, however, a common type of decision we make every day is determining how much time it is worthwhile to spend on one activity before switching to another. Effectively managing time presents a complex cognitive challenge, especially in the context of addictive behaviors, depression, and the pervasive influence of technologies designed to capture attention for prolonged periods. Here, we aimed to investigate the neural mechanisms underlying real-time monitoring of activity values and strategies for deciding when to disengage from pursuits with diminishing returns.

### 1.1 Ecological Models of Time Allocation

These types of continuous, temporal decisions are common within ecological foraging paradigms. Specifically, patch foraging presents animals with an environment where resources are clustered in patches. The utility of searching a patch declines over time as its resources are harvested, requiring the animal to decide when to abandon it in search of a new one. Many ethological studies have characterized various factors that affect patch occupancy time, such as patch richness (Keller & Sullivan, 2023; Marshall et al., 2013; Utsumi et al., 2009), inter-patch distance (Searle et al., 2006; Utsumi et al., 2009), predation risk (Brown, 1988; Eccard et al., 2020; Ward et al., 2000), and competition (Brown, 1988; Laguë et al., 2012). To understand how long an animal should continue searching a given patch, we use the normative principle of reward-rate maximization, which posits that the best strategy is the one that results in the highest overall rate of reward. For typical patch-foraging tasks, where the patch’s reward probability decays monotonically over time, the optimal policy is to leave a patch when the rate of reward inside the patch falls below the overall rate of reward achieved in the environment, as delineated by the Marginal Value Theorem (MVT) (Charnov, 1976). The MVT provides the mathematically optimal time to exit a patch, yet patch occupancy across organisms is consistently reported to be overpatient compared to optimal (Baum, 1983; Bettinger & Grote, 2016; Güldener et al., 2024; Hall-McMaster et al., 2021; Harhen & Bornstein, 2023; Z. Kilpatrick et al., 2020; Lloyd et al., 2023; Marshall et al., 2013; Nonacs, 2001; Wajnberg et al., 2000).

### 1.2 Neural Substrates of Decision-Making and Timing

The basal ganglia play a key role in action selection and option evaluation during decision-making (Cisek, 2007; Mink, 1996; Redgrave et al., 1999). The primary input nucleus of the basal ganglia, the striatum, receives cortical projections from a wide variety of regions, integrating information about current goals and shifting environmental variables to guide behavior (Balleine et al., 2007; Ferenczi et al., 2016; Frank et al., 2004). Subregions of the striatum engage with different aspects of optimizing behavior, with the ventral striatum processing reward-related information (Berridge & Robinson, 2003; Salamone & Correa, 2012), the dorsolateral striatum mediating habit formation (Packard & Knowlton, 2002; Smith & Graybiel, 2013; Yin et al., 2004), and DMS engaged in goal-directed decision-making (Balleine et al., 2007; Yin et al., 2005). Lesions of DMS severely impair behavioral flexibility in animals, causing them to lose their ability to adapt to shifting action-outcome contingencies (Castañé et al., 2010; Pisa & Cyr, 1990; Ragozzino, 2007; Torres et al., 2016; Yin et al., 2005), while electrophysiological recordings show that DMS neurons dynamically encode decision variables, including action values, predicted outcomes, and choice signals (Burton et al., 2015; Ito & Doya, 2015; Kim et al., 2013; Lau & Glimcher, 2008; Parker et al., 2016; Thorn et al., 2010).

However, much of the decision-making research in DMS uses tasks in which subjects choose between discrete options, such as two-alternative forced choice and multi-armed bandit tasks (Balleine et al., 2007; Bogacz et al., 2006; Samejima et al., 2005; Stott & Redish, 2014; Tang et al., 2022). Patch foraging presents a different type of decision where the choice measured is a continuous variable. Rather than deciding *what* action to take, subjects must determine *when* to take action. The striatum has also been strongly implicated as a region central to timing, with lesions causing impairments in interval timing and temporal discrimination (Matell & Meck, 2004; Meck, 2006; Merchant et al., 2013). DMS neurons have been shown to encode temporal information, through activity patterns such as ramping (Emmons et al., 2017; Ponzi & Wickens, 2022; Vandaele et al., 2021), and temporal receptive fields (Gouvea et al., 2015; Jin et al., 2009; Mello et al., 2015). The convergent role of the DMS in both reward-related decision-making and interval timing makes it well-suited to meet the temporal decision-making requirements for efficient patch foraging behavior.

Dopamine projections to the striatum have also been implicated in both timing research and reward-related decision-making. Dopamine is a substantial modulator of both medium spiny neurons and cholinergic interneurons (Cai & Ford, 2018; Chen et al., 2021; Clarke & Adermark, 2015; Gagnon et al., 2017) and has been shown to be critical for learning and updating the action-outcome associations that guide goal-directed behaviors (Baudonnat et al., 2013; Berke, 2018; Blanco-Pozo et al., 2021; Guru et al., 2020; Peak et al., 2019; Roesch et al., 2007). During interval timing, manipulations of DMS dopamine affect the duration of timed intervals, where the level of dopaminergic signaling is proposed to modulate internal clock speed (Fung et al., 2021; Soares et al., 2016). DMS dopamine is also implicated in foraging-like decisions, with pharmacological results suggesting that it carries contextual information that affects an animal’s decision of when to leave the current patch (Le Heron et al., 2019). This positions DMS dopamine as a neural substrate that may convey information about the moment-by-moment reward landscape of a foraging patch.

### 1.3 Study Overview and Objectives

To understand how the action selection and timing aspects of DMS activity work together to promote efficient patch-foraging behavior, we set out to characterize both single-cell activity and broad dopaminergic signaling in the DMS during a patch-foraging task developed for freely moving mice. Trained mice were either implanted with Neuropixel probes or injected with a virus containing GRAB-DA3m, a dopamine sensor, and implanted with photometry fibers to observe neural and dopaminergic activity, respectively, leading up to the decision to exit a patch.

We found that mice adopted a reward-reset strategy, primarily basing their patch exits on the time since the most recent reward, combined with effects from patch occupancy time and the reward-rate context. The longer the mouse had already spent in the patch, the less time they would be willing to wait for the next reward. The time the mouse would wait following a reward similarly decreased when the overall reward rate of the environment was high, reflecting an increased cost of time. Interestingly, the task was designed such that the optimal time to leave the patch was independent of individual reward events, yet mice exhibited a consistent preference for timing their exits from the most recent reward. Neurally, ensembles of DMS neurons timed the interval between each reward and the moment the mouse would exit if no more rewards were delivered. Individual neurons underwent transitions between high and low activity states following rewards, with preferred transition times that spanned the duration of the intended interval. At the population level, the number of transitioned units accumulated linearly following rewards, reaching a threshold as the mouse exited the patch. The slope of the accumulation reflected the mouse’s intended exit time, accumulating faster when the overall reward rate was high and after longer patch occupancy times. Dopaminergic transients followed each reward, encoding reward prediction errors that correctly conveyed the underlying reward probability. Together, these findings suggest that the DMS plays a key role in regulating temporal decision-making during reward-seeking behavior.

## 2. Results

### 2.1 Behavioral Characterization of Patch-Foraging Strategy

**Figure 1:**
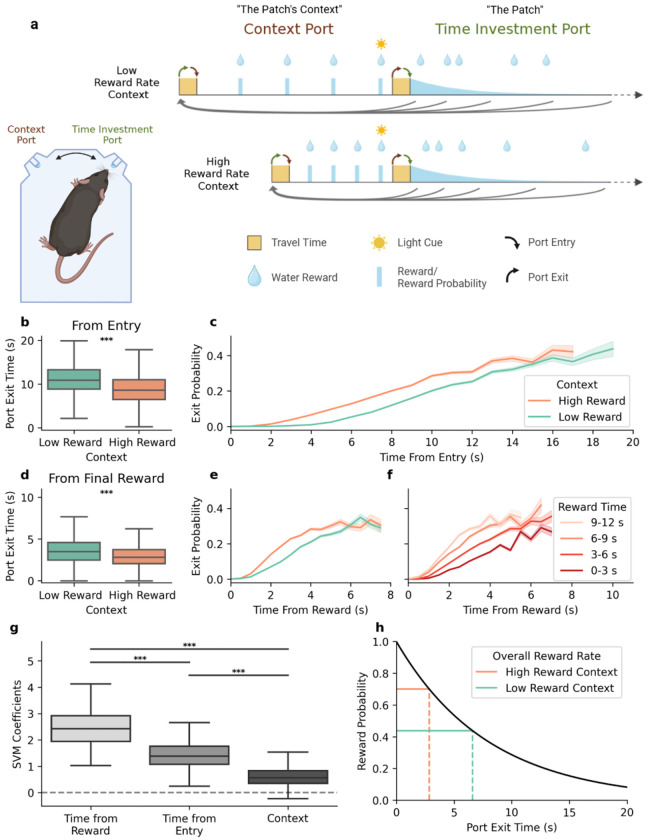
Task structure and behavioral strategy. **a)** Schematic of the behavioral box (left) and structure of the task (right). Mice alternate between collecting rewards from the “context port” and the “time-investment port”. In the context port, four rewards are delivered at regular intervals before the port becomes inactive, indicated by a light cue turning on. In the time-investment port rewards are delivered stochastically with exponentially decreasing probability. The context port is reset when the mouse enters the time-investment port, while the time-investment port is reset after the mouse receives all four rewards in the context port. **b)** Observed exit times from the time-investment port across mice (n=18), measured from the time of entry into the port. **c)** The probability (hazard rate) of exiting the time-investment port based on the time since port entry (x-axis) and the context port reward rate (hue). **d)** Same as **b**, except measured from the time of the final reward received in the time-investment port. **e)** The probability (hazard rate) of exiting the time-investment port based on the time since the most recent reward (x-axis) and the context port reward rate (hue). **f)** The probability (hazard rate) of exiting the time-investment port based on the time since the most recent reward (x-axis) and the time of the most recent reward relative to time of port entry (hue). **g)** Support vector machine (SVM) coefficients for predicting time-investment port occupancy. **h)** The theoretically optimal times to exit the time-investment port (dashed vertical lines) for each context block based on the maximum achievable overall reward rates (solid horizontal lines) in accordance with the Marginal Value Theorem (MVT).

We began by characterizing time investment behavior on a patch foraging task that required subjects to decide how long to stay in a patch under shifting environmental conditions. Mice were placed in custom behavioral chambers with access to two nose-poke ports, the ‘time-investment port’ and the ‘context port’ (Figure 1a). The time-investment port served as the ‘patch’, where water rewards were randomly delivered from a lick spout with an exponentially decreasing probability over time. Mice invested time in the patch by maintaining their heads in the time-investment port, licking continuously as they harvested water rewards. As the likelihood of reward decreased with time, mice could choose to abandon the time-investment port at any moment in favor of the context port. The context port provided a fixed number of regularly spaced rewards over a fixed interval, after which a light cue turned on to indicate that the context port was depleted and the time-investment port had been replenished. As soon as the mouse returned to the time-investment port, the light cue turned off, signaling that the context port was replenished and the mouse could again decide to leave the time-investment port at any point. Mice learned to alternate back and forth between the two ports throughout each behavioral session, investing a self-determined amount of time in the time-investment port.

Sessions were broken into blocks where the context port switched between being ‘high’ value (four rewards in five seconds) and ‘low’ value (four rewards in ten seconds), while the reward probability distribution in the time-investment port remained constant (Figure 1a). Critically, this experimental design shifted the reward-rate optimizing patch-exit strategy solely by manipulating factors external to the patch (Figure 1h). The central behavioral measure of interest was the amount of time invested in the time-investment port during each trial. We found that, on average, mice invested more time in the time-investment port during ‘low’ context port blocks compared to ‘high’ context port blocks (Figure 1b), progressively increasing their likelihood of departure as time passed in the time-investment port (Figure 1c). While the direction of this context-dependent shift aligns with theoretical expectations (Charnov, 1976; McNamara & Houston, 1985; Sutlief et al., 2025) and prior observations (Constantino & Daw, 2015; Z. P. Kilpatrick et al., 2021; Marshall et al., 2013), the average patch occupancy time per trial exceeded optimal, a trend well-established in previous work (Constantino & Daw, 2015; Hayden et al., 2011; Hutchinson et al., 2008; Lenow et al., 2017; Nonacs, 2001; Wilke et al., 2009).

### 2.2 Reward-Reset Exit Strategy

Since the probability of reward depended only on the time since entering the port, the optimal departure time is not affected by the specific reward events on individual trials. Nonetheless, the departure strategy exhibited by mice was very sensitive to the timing of rewards received in the time-investment port. While mice experienced sufficient training to be abundantly familiar with the underlying reward probability distribution, the time since the previous reward was the strongest factor determining port exit (Figure 1g). To better understand how reward events shaped exit policy, we examined the hazard rate of leaving the patch during the interval following rewards, split by context (Figure 1e) and the time of the reward relative to port entry (Figure 1f). We found that all three of these factors contributed to exit behavior, where each reward received would “reset” the amount of time the mouse would be willing to wait to a duration determined by the patch occupancy time thus far and the environmental reward-rate context.

### 2.3 DMS Neurons Undergo Firing Rate State Transitions Following Rewards

**Figure 2:**
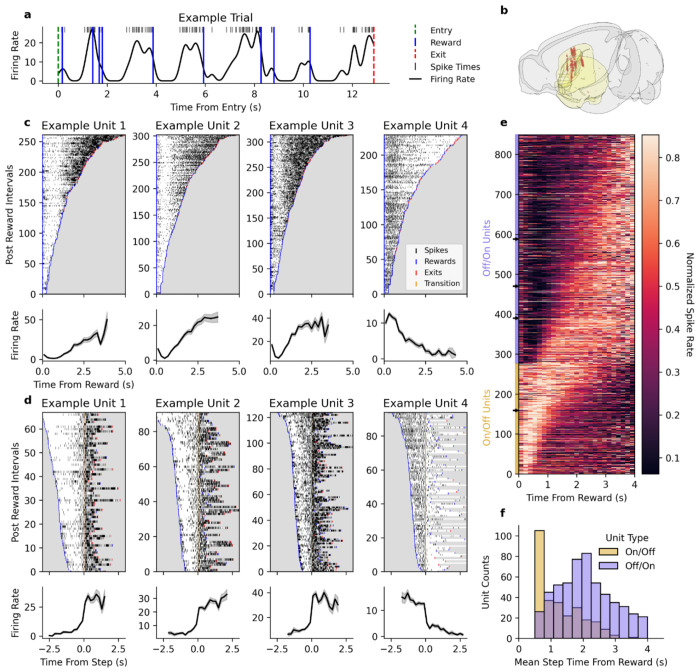
Dorsomedial striatum neurons exhibit step-like activity patterns following rewards. **a)** Example activity of a recorded unit during a single trial in the time-investment port. Top: Example unit spike raster. Bottom: Firing rate trace calculated by binning spikes (10 ms bins) and smoothed with a Gaussian kernel (σ = 100 ms). **b)** Anatomical localization of recorded units in DMS for all recorded neurons (1,845 units across 10 mice). **c)** Four example units showing reward responsive patterns. Top: Spike rasters for each reward-reward and reward-exit interval, ordered by the duration of the interval. Bottom: Corresponding mean firing-rate traces aligned to reward delivery (time = 0). **d)** Same as **c**, except showing intervals where each unit exhibits a state-transition, a discrete step in activity post reward, identified by the inflection point of a sigmoid function fit to the firing rate. Rasters and mean firing rate traces are aligned to the detected step times (yellow). **e)** Heatmap showing the normalized firing rates of all DMS units that make step transitions following rewards. Units are ordered by the direction of their activity step and their mean step time. Example units are marked with arrows. **f)** Distribution of the mean activation times of the units displayed in **e**.

Neural activity in DMS was similarly sensitive to rewards. Figure 2a shows activity from a single neuron on a single trial, revealing a pattern of suppression, followed by activation, that was prevalent within the recorded population. The activity of four example neurons, aligned to rewards in the time-investment port, is shown in Figure 2c, revealing what appear to be ramping signals in the activity traces averaged across rewards (Figure 2c, bottom row). However, responses following individual rewards show discrete jumps between high and low firing rates, which becomes evident when aligning to the transition time instead of the reward (Figure 2d). We developed a method to classify neurons’ responses as step-like and to determine the precise moment of each transition (see methods). Out of the 1,845 identified neurons, a total of 849 were lawfully categorized (see methods) with this reward-triggered state-switching activity pattern. The normalized activity of these neurons following rewards is shown in Figure 2e, ordered by their average step time. The majority of neurons exhibited suppressed firing immediately following rewards, then activated after a delay (off/on units), but another portion showed the opposite (on/off units). The distribution of average step times within these two groups differed, with on/off units clustered at the beginning of the interval (Figure 2f). Together, the step times are distributed across the interval following rewards, forming a representation of time that can be read out by measuring the accumulation of transitioned neurons.

### 2.4 Neural Step Times Correlate with Behavioral Exit Policy

**Figure 3:**
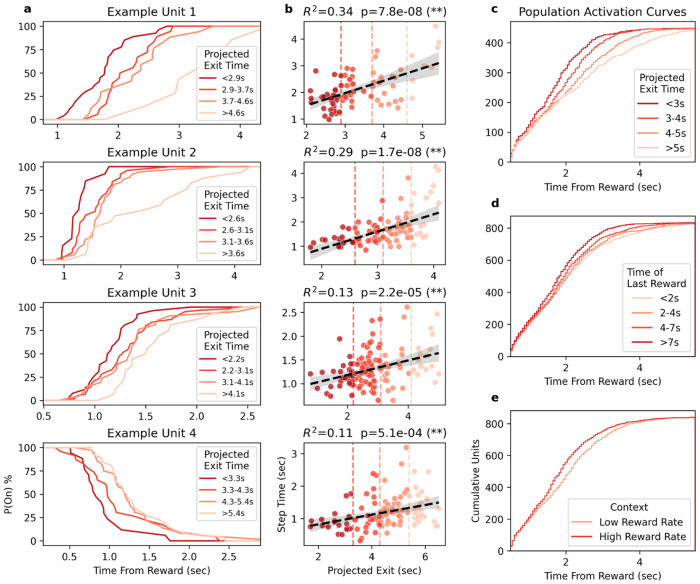
Neural state-transition times correlate with projected exit times. **a)** Plots for each example DMS unit (same as Figure 2c,d) showing the probability of the unit being in the “on” state in the time following reward, separated by quartiles of the projected behavioral exit time (See Supplemental Figure 2). **b)** Correlation between unit state-transition times and projected exit times for each example unit. Hue and vertical dashed lines indicate the quartile groupings used in **a**. **c)** Population mean state-transition times shown as a cumulative function for each of the four projected exit time ranges, including only units with measurements in every time range. All groups differ significantly (Kolmogorov-Smirnov, all p < 0.05). **d)** Same as **c**, except separated by the time of the last reward. Comparisons “<2s” vs “>7s” and “2-4s” vs “>7s” differ significantly (Kolmogorov-Smirnov, p < 0.05), all group comparisons are not significant. **e)** Same as **c**, except separated by the reward rate in the context port. Groups differ significantly (Kolmogorov-Smirnov, p < 0.05).

While the exit time policy across all sessions and subjects has a relatively high variability, the policy within individual sessions is far more consistent (Supplemental Figure 1a). We used a simple linear regression model to predict expected exit times for each reward, if that reward had been the last to be delivered in the time-investment port during that trial (see methods section 4.10, Supplemental Figure 2). For the four example neurons in Figure 3, we plotted the probability of the neuron being in the “On” state during the period following rewards, dividing the rewards into groups by projected exit time (Figure 3a). We found that each example neuron transitioned later when the mouse was expected to exit later, and earlier when the mouse was expected to exit earlier. Plotting their transition times against the projected exit times, we observe a linear correlation (Figure 3b). Extending this analysis to the rest of the step-like neurons, we find that neurons with later average step times correlate more strongly with the projected exit time, while neurons with earlier average step times correlate a smaller amount or not at all (Supplemental Figure 5). To examine this trend across the population, we plotted the post-reward accumulation of neuronal state transitions based on average transition times for four projected-exit time bins (Figure 3c). Since this visualization is an aggregation of data across sessions with variable exit policies, the time bins don’t capture all of the variability within individual neuron transition times, yet the general trend of larger shifts for neurons with later transition times may be observed. Separating the groups by the time of the reward relative to port entry shows a diminished separation, where only a subset of group comparisons are significantly different (Figure 3d, see Figure 3 legend for all stats). Separating by reward-rate context also shows a diminished, though still significant, difference (Figure 3e).

### 2.5 Accumulation of Neural State Transitions Predicts Exit Time

**Figure 4:**
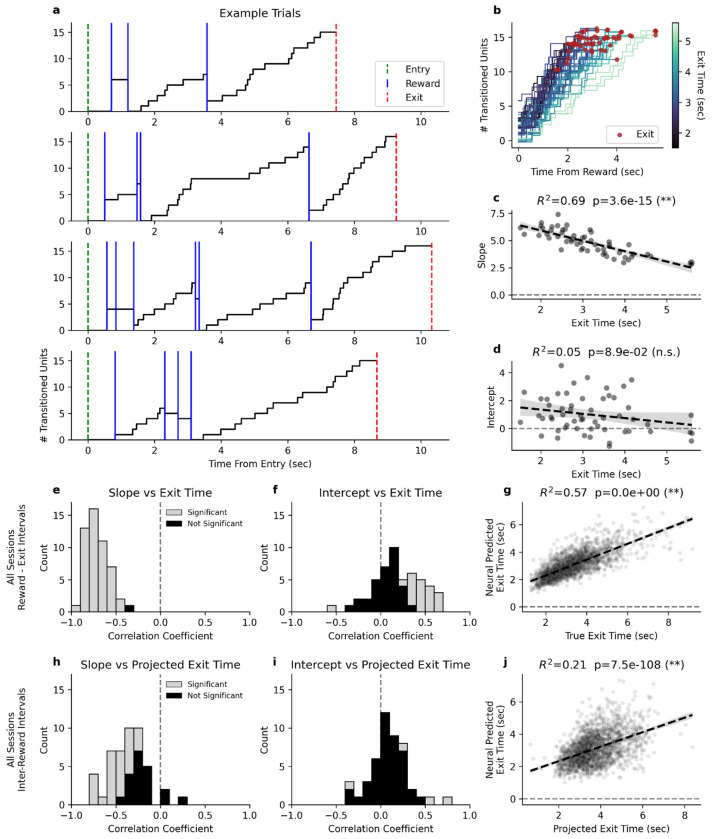
Accumulation of neural state transitions in DMS predicts exit timing. **a)** Four example trials from a single session, showing the cumulative step transitions of simultaneously recorded units. Vertical lines indicate entry (green), exit (red), and reward events (blue). **b)** The accumulation lines for all of the reward-to-exit intervals from the same session as the examples in **a**. The lines are colored based on the duration of time between the reward and the exit events. Exits are marked by maroon dots capping each line. **c)** The slope of each accumulation line in **b** fit with a linear model, plotted against the exit time. **d)** The intercept of each accumulation line in **b** fit with a linear model, plotted against the exit time. **e)** A histogram of the correlation coefficients between the accumulation slopes and exit times for all sessions with more than 6 step-like units. **f)** A histogram of the correlation coefficients between the accumulation y-intercepts and exit times for all sessions with more than 6 step-like units. **g)** The relationship between the exit time predicted by the neural activity based on an accumulate to threshold model and the true exit times for all reward-to-exit intervals. **h)** A histogram of the correlation coefficients between the accumulation slopes and behaviorally projected exit times during reward-to-reward intervals for all sessions with more than 6 step-like units. **i)** A histogram of the correlation coefficients between the accumulation y-intercepts and behaviorally projected exit times during reward-to-reward intervals for all sessions with more than 6 step-like units. **j)** The relationship between the exit time predicted by the neural activity based on an accumulate to threshold model and the behaviorally projected exit times for all reward-to-reward intervals.

To understand how the accumulation of state transitions related to exit behavior, we examined the population activity of simultaneously recorded neurons. We identified 53 sessions with sufficient step-like units to quantify population-level dynamics. Figure 4a shows several representative trials from an example session in which 16 step-like units were detected. Following rewards, units steadily transitioned to their post-step state, producing an accumulation of transitioned units over time, which reset with each reward. When considering all the reward-to-exit intervals (Figure 4b), we observe that the accumulation of transitioned units ramps more quickly when the mouse is to exit earlier and more slowly when the mouse is to exit later. Leading up to every exit, this neural population accumulated to around 15 transitioned units, suggesting a threshold mechanism that could govern patch exit behavior. By fitting linear functions to the accumulation of units for every reward-to-exit interval, we found that the slope of accumulation was significantly negatively correlated with the exit time (Figure 4c), while the intercept was not significantly correlated (Figure 4d).

When performing this analysis for every session, we observed that the slopes were significantly negatively correlated with exit times for all but one session (Figure 4e), while the intercepts displayed weak or nonexistent correlations with exit times (Figure 4f). By using accumulation thresholds to predict when the mouse would exit, we generated neurally predicted exit times that were significantly correlated with the true exit times (Figure 4g). We also calculated fit lines for the accumulation of step-like transitions on all the inter-reward intervals (excluding reward-exit intervals) and compared them with behaviorally projected exit times, finding similar correlation patterns (Figure 4h-j).

### 2.6 Dopamine Transients Encode Reward Prediction Error Signals

While both our neural data and behavioral observations show timing based on the most recent reward, this is in conflict with reward-rate optimal behavior. Since the probability of reward at any given moment within the time-investment port is independent of any other moment, an optimal agent would exit at a fixed amount of time after entering the port, regardless of the occurrence and timing of rewards experienced on any given trial. This raises the possibility that mice are learning reward-rate suboptimal value-time functions that are structured on the reward events instead of the time of entry of the time-investment port.

**Figure 5:**
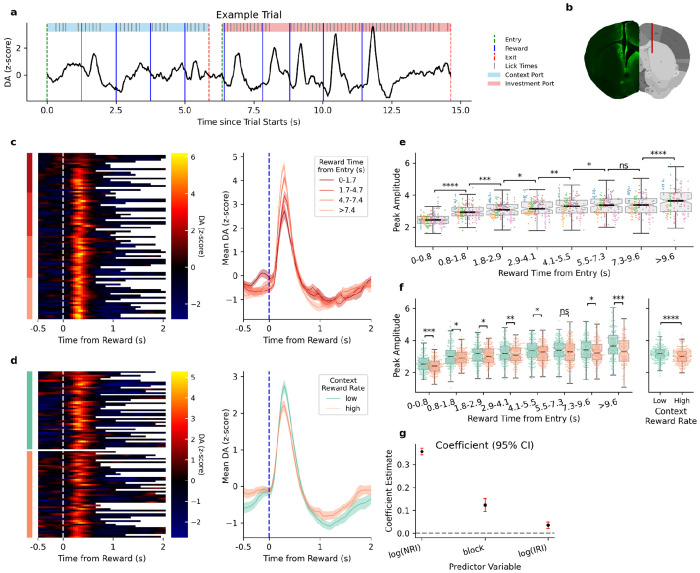
Dopamine in DMS encodes reward timing and context. **a)** Example DMS dopamine trace (z-score) from a single behavioral trial. The mouse first waits in the context port (blue shading) consuming its regularly spaced rewards before moving to the time-investment port (pink shading) to wait for its rewards, delivered with an exponentially decreasing probability. Vertical lines indicate port entries (green), exits (red), and rewards (blue). Individual licks are marked by short vertical ticks. **b)** Histological verification of GRAB-DA sensor expression and optical fiber placement in the DMS. The left half shows a representative slice with the fiber track and fluorescence from antibodies that bind to the sensor; the right half shows the planned fiber targeting (red line) on a schematic from Allen Brain Atlas. **c)** Dopamine dynamics from an example session, sorted by reward time from entry. Left: Heatmap of dopamine activity aligned to reward delivery. Trials are sorted by the time from port entry to reward. The color bar on the left indicates quartiles of reward time. Right: Average dopamine traces for each quartile. The shaded error band represents the standard error of the mean (SEM). **d)** Dopamine dynamics from an example session, separated by context block and sorted by reward time from entry. Left: Heatmap of reward-aligned dopamine activity, separated by low (green) and high (orange) reward-rate contexts. Right: Average dopamine traces for low and high reward-rate contexts (shaded band is SEM). **e)** Dopamine peak amplitude vs. reward time from entry, across all animals and sessions. For each session, rewards were grouped into time windows after port entry. Each data point is the average dopamine peak amplitude for all rewards within one of those windows of a single session. Boxplot shows the median and quartiles of dopamine peak amplitude across all animals and sessions. Colors of the data points indicate animals (n=8), with 10-20 sessions acquired from each animal. Paired t-tests show significant differences between most neighboring time windows. **f)** Left: Dopamine peak amplitude as a function of reward time from port entry, separated by low (green) and high (orange) reward-rate contexts. Rewards were grouped into time windows, with each data point representing the session-average DA peak amplitude for all rewards within one context and time window. Boxplots show the median and quartiles across all animals and sessions. Paired t-tests show significant differences between contexts within most time windows. Right: Dopamine peak amplitude across context blocks, averaged per session. Colors as above. Paired t-tests show significant differences between the two context blocks. **g)** Linear mixed-effects model estimates for the effects of three environmental features on dopamine peak amplitude. Points represent the fixed-effects coefficients for each predictor, and vertical lines denote the 95% confidence intervals (CIs). The model predicted dopamine peak amplitude as a function of reward time from entry, context block reward rate, and inter-reward interval.

To assess the putative value learning signals received by these neurons during this task, we used fiber photometry to measure dopamine levels in DMS. We observed large dopamine transients that peaked approximately 300 ms after rewards in the time-investment port (Figure 5a). The amplitude of the reward-related transients correlated with the time since port entry, where rewards that occurred later within the time-investment port evoked larger dopamine transients (Figure 5c/e). The change in magnitude by time in port is consistent with reward prediction error signaling, with larger responses evoked by rewards occurring later in the patch despite their lower probability. The reward-rate context also modulated the amplitude of the dopamine transients, with larger transients occurring during the low reward-rate blocks (Figure 5d/f). As rewards are sometimes delivered in quick succession (down to 100ms apart), we observed a suppressive effect of recent rewards on DA transient amplitude (Figure 5g). Rewards delivered within one second of a previous reward evoked a smaller DA response, but with inter-reward intervals longer than one second, we observed little effect of inter-reward interval on DA transient amplitude.

## 3. Discussion

Our findings reveal a novel mechanism by which DMS may support temporal decision-making during patch-foraging behaviors. We demonstrated that mice adopt a scaling reward-reset strategy, where they exit the patch a predictable amount of time after reward, decreasing linearly with patch occupancy time and shifting inversely with the environmental reward rate. This temporal structuring of behavior is mirrored in the two-state firing rate dynamics of DMS neurons, which undergo state transitions at variable delays following rewards. We showed that the timing of the state transitions was correlated with patch exit times and that populations of simultaneously recorded neurons linearly accumulated state transitions up to a threshold coinciding with patch exit. Together with our observations of reward prediction error-like dopaminergic signaling, these results show how striatal circuits can integrate timing information and reward expectations, leading to flexible decision-making in naturalistic foraging contexts.

### 3.1 Neural Mechanisms of Temporal Decision-Making

At the individual neuron level, we observed step-like responses in DMS neurons, differing from the ramping or sparse temporal response dynamics typically associated with timing processes. Previous studies on interval timing in the striatum have predominantly reported firing rates that increase steadily over the course of the interval (Gouvea et al., 2015) or show sparse encoding of time fields (Mello et al., 2015). At the population level, our findings indicate that the striatum exhibits activity patterns that accord with the curious features of the behavioral strategy employed, where the intended interval to depart from a patch appears as timed from the last reward, though modified by the context in which the patch is embedded, and by the amount of time already spent in the patch. Specifically, the number of transitioned units resets and accumulates linearly after each reward, with the slope of this accumulation predicting the actual exit time from the patch. As with behavior, the time already spent in the patch as well as the patch context impact timing, affecting the rate of accumulation of transitioned units. When rewards occur earlier in the patch the slope of accumulation is shallower, predicting greater time investment, whereas if rewards occur later in the patch, the slope of accumulation is steeper, predicting early departure times. When the time spent outside the patch in the context port is greater, and the reward rate of the context port lower, the cost of time is low, resulting in a greater willingness to invest time in the patch. This is reflected in the correspondingly lower rates of accumulation of transitioned units in comparison to when the patch’s context is ‘high’. Collectively, these observations indicate a neural mechanism in which the DMS conveys the intended waiting interval after a reward, integrating elapsed time since last reward, elapsed time since patch entry, and the context of the patch.

### 3.2 Reward-Reset Strategy and Behavioral Optimality

In contrast to the behavioral predictions of classical optimal foraging theory, we observed the use of a reward-reset strategy, where an outsized emphasis was placed on the timing of the most recent reward. According to the Marginal Value Theorem, the optimal patch departure time occurs when the instantaneous reward rate falls below the environmental average (Charnov, 1976), where the timing of individual rewards in a fully stochastic environment should have no effect. However, our mice consistently used the time since the most recent reward as their main decision variable, similar to the strategies previously observed in parasitic wasps (Haccou et al. 1991; van Alphen et al. 2003; Wajnberg et al. 2000). Other variables, such as the reward-rate context and time spent in the patch, had modest but significant effects on the time to exit following each reset, demonstrating sensitivity to the time-evolving value of the patch, and to the environmental reward-rate context.

The suboptimality we observed (mice staying in patches longer than predicted by MVT) is consistent with previous reports of “over-patience” in patch-foraging tasks (Constantino & Daw, 2015). This pattern may reflect some inherent cognitive constraints in temporal decision-making (Cuthill et al. 1990; Kacelnik and Krebs 1985; Krebs et al. 1974) or could represent an adaptive response to environmental uncertainty (Kacelnik and Todd 1992; Kacelnik and Bateson 1996; Olsson and Brown 2006), where a longer occupancy time allows for more information collection before a certainty threshold is crossed. The reward-reset strategy may be particularly advantageous in environments where patch quality varies unpredictably, since recent rewards would provide more reliable information about the patch’s current reward rate than a previously accumulated estimate of environmental statistics. However, this is notably not the case for the task described in this study.

### 3.3 Dopaminergic Signaling and Value Learning

The dopaminergic responses measured in the DMS showed a pattern akin to reward prediction error signaling, with larger transients following rewards that occurred later relative to patch entry when the reward probability was lower. These transients were also enhanced during low reward-rate blocks compared to high reward-rate blocks. The directions of these two shifts are inconsistent with each other for a clock speed modulation hypothesis, where dopamine signals would directly affect temporal processing speed to drive patch exits. Under such a mechanism, the clock speed should be faster during high reward-rate context blocks and when rewards are delivered later, yet dopamine transients are modulated in opposite directions for these two conditions. This dissociation between dopamine amplitude and behavioral timing suggests that these signals do not directly modulate the rate of temporal processing in individual trials.

Rather, our findings are consistent with dopaminergic encoding of relative reward prediction errors, where transients reflect the contextualized value of rewards within current environmental conditions. Similar to classical reward prediction errors, dopamine transients are larger following rewards that occur later within the time-investment port despite a progressively decreasing probability. While the magnitude of reward prediction errors in the time-investment port should be the same between contexts, all state values would be lower during the low reward-rate context, so the reward prediction error relative to the state value would be larger.

This interpretation suggests that dopaminergic signaling in the DMS serves to maintain a representation of the time-investment port’s reward density relative to environmental conditions rather than directly driving moment-to-moment temporal decisions. Despite mice not using that representation to directly determine their exit time, as would be consistent with optimal foraging theory, their representation of the underlying reward rate influences the amount of time they are willing to wait following each reward. Such an effect may be mediated by dopamine through the long-term modulation of synaptic connections in the striatum (Calabresi et al., 2007; Gerfen & Surmeier, 2011; Kreitzer & Malenka, 2008; Surmeier et al., 2009; Wickens, 2009), allowing the system to remain sensitive to any future shifts in the underlying reward probability.

### 3.4 Conclusion

Taken together, our results suggest that neural population activity in DMS dynamically encodes intended action intervals during patch leaving behavior. The rate of accumulation of neural state transitions scales to span the duration of intended reward-to-exit intervals. This may represent the integration of influences from a wide variety of environmental factors that shape efficient foraging behavior. We suggest that DMS plays a key role in forming time-investment strategies that weigh the value of outcomes against the time it takes to achieve them.

## 4. Methods

### 4.1 Compliance Statement

All animal procedures were conducted in accordance with the National Institutes of Health guidelines and were approved by the Johns Hopkins School of Medicine Institutional Animal Care and Use Committee (IACUC).

### 4.2 Subjects

Ten C57BL/6J mice (4 male, 6 female) were used in the electrophysiology component of this study, while eight C57BL/6J mice (4 male, 4 female) were used in the fiber photometry component. Mice were purchased from The Jackson Laboratory and housed in standard vivarium cages. All animals had *ad libitum* access to food and water, except during periods of behavioral training and electrophysiological recording, when water was restricted. All animals maintained 85-90% of their original body weight throughout water restriction.

### 4.3 Task Design

Mice were trained on a patch-foraging task designed to specifically observe and measure the moment when a mouse decides to give up on a depleting patch (Figure 1a). The timing of that decision optimally depends on the relative expected value of remaining in the patch compared with the overall expected value of the environment. In our task, mice are required to harvest from a patch under low and high reward-rate environments, while the underlying reward probability function within the patch remains consistent. Broadly, the task requires the mouse to switch back and forth between two ports as it harvests water rewards. The two ports are the time-investment port, which functions as a patch with reward delivered stochastically according to an exponentially decaying distribution, and the context port, which delivers four evenly spaced rewards over either a short, or a long interval, depending on block.

The task is performed in a custom-built 7” x 5” box where the mouse has access to two ports it may poke its head into. The ports are equipped with lick spouts that deliver 1 μL water rewards, infrared sensors that detect head entry and licks, and LED light indicators that signal whether or not the port is active (LED off = port is active/rewards are available, LED on = port inactive/rewards are not available). The mouse begins in the first of the two ports, the context port, where it receives 4 water rewards regularly spaced over either 5 or 10 seconds, depending on the current block. After the final reward is delivered, the context port light turns on and the time-investment port light turns off, indicating the mouse may then switch ports. Travel between the ports takes approximately 300 ms. Upon entering the time-investment port, the context port light turns off, signalling that the mouse may abandon the time-investment port in favor of the predictable context port rewards at any time. For as long as the mouse chooses to stay, the time-investment port delivers rewards probabilistically, with the likelihood of reward decreasing exponentially as time passes. The cumulative probability of the exponential decay curve sums to eight, meaning that on average, staying indefinitely in the time-investment port would yield eight rewards, while the actual number of rewards that can be delivered is unbounded. Eventually, the moment of experimental interest arrives, being when the mouse decides to exit the time-investment port and return to the context port. As soon as it reenters the context port, the time-investment port light turns on and a single cycle (trial) is complete. Each time the mouse consumes a set of four context port rewards, the time-investment port reward probability is refreshed to its starting value, allowing the mouse to continuously harvest by alternating between the ports.

To consider a mouse fully trained, we required that they did not leave the context port prematurely on more than 20% of the trials and that they move to the time-investment port within a few seconds of the light cue turning on in the context port. This ensured that they experienced the intended reward rate when deciding when to leave the time-investment port. No requirements are placed on their strategy in the time-investment port.

Each behavior session lasted 18 minutes and was divided into six three-minute blocks. Across blocks, the dynamics of the context port alternated between providing a high reward-rate context (4 rewards in 5 seconds) and a low reward-rate context (4 rewards in 10 seconds). The value of the starting block (‘low’ or ‘high’) was randomly determined for each session. The reward probability distribution of the time-investment port remained consistent throughout all trials across all mice. All of the logic of the task, as well as control of the sensors, LEDs, and solenoids, was custom written in python and deployed on a Raspberry Pi. All time stamped events were saved to a text file for later analysis.

### 4.4 Analysis - Support Vector Machine (SVM) Model

To understand the factors contributing to the decision of when to leave the Time-Investment Port, a Support Vector Machine (SVM) classifier was used to identify the conditions under which a mouse would either stay or leave. Three factors were supplied to the SVM: The time since entry into the Time-Investment Port, the time since the most recent reward in the Time-Investment Port, and the reward-rate context (either low or high). Each trial from a given session was divided into 10 ms time bins and assigned to the ‘Stay’ group. The five seconds following each exit was similarly divided and assigned to the ‘Leave’ group. The SVM then used a linear kernel to calculate the plane that best separated the two groups. The coefficients of each normalized feature were then compared across sessions to determine the relative importance of the time from reward, the time from entry, and the reward-rate context to the patch-leave decision strategies used by mice across sessions.

### 4.5 Surgery for Neuropixel Implantation

Mice underwent stereotaxic surgery to implant Neuropixel 2.0 (multi-shank) probes using a custom 3D printed headcap and probe insertion assembly. All surgeries were performed in aseptic conditions and followed an IACUC approved protocol. Animals were anesthetized with isoflurane and their body temperature was maintained at 37°C. Ophthalmic ointment was applied to the eyes and Lidocaine (0.0021 mL/g) was injected subcutaneously under the scalp. Fur was removed from the scalp before it was disinfected with alternating Betadine and 70% isopropyl alcohol. A midline incision exposed the skull, with excess skin removed to expose a large surface area of skull to ensure the headcap could be securely attached. The periosteum was cleared with alternating 3% hydrogen peroxide and 70% isopropyl alcohol. The skull was then leveled and the craniotomy target (dorsomedial striatum, AP: 0.7 mm, ML: 1.2 mm) was measured from bregma. The skull surface was thoroughly dried and a thin layer of Metabond dental cement was applied to the entire exposed surface, which was given 10 minutes to fully set. The craniotomy target was then marked on the metabond, and an oval section (approximately 1.2 mm x 1 mm) was drilled through both metabond and skull. A separate small hole was drilled for a ground pin posteriorly on the contralateral side. A small section of dura was carefully removed from the craniotomy. The custom headcap was then placed over the craniotomy, with an oval lip fitted inside to prevent skull regrowth around where the Neuropixel probe would be inserted. A gold grounding pin was placed in the posterior hole. Metabond was used to secure both headcap and pin to the skull, ensuring the craniotomy and ground pin hole were fully enclosed. Buprenorphine (0.0083 mL/g) was then administered subcutaneously on the lower back, approximately 5 minutes prior to the end of the surgery. The Neuropixel 2.0 probe was then slowly inserted into DMS, using a built-in screw and shuttle system custom designed to interface with the headcap. The probe was lowered to a target depth of 5mm at a rate of 1 mm/min. The ground pin was connected to the grounding pad of the Neuropixel probe via a soldered silver wire and the Neuropixel housing assembly was secured to the headcap with two external screws. The weight of the entire piece reached approximately 3 grams. Mice were supplied with hydration gel and monitored as they recovered in a heated cage until they were fully awake.

### 4.6 Surgery for Viral Injection and Photometric Fiber Implantation

Surgical protocol for the craniotomy preparation was the same as in Neuropixel Probe implantation. For viral injection, a Nanoject microinjector, fitted with a glass micropipette containing the diluted viral vector, was positioned over the center of the craniotomy and lowered to a depth of 2.3 mm below the dural surface. Adeno-associated virus (AAV9) packaged with hsyn-GRAB-DA3m (Zhuo et al., 2024) (WZ Biosciences; titer > 10^13 gc/ml) was mixed 1:1 with saline. A total volume of 300 nL of viral solution was injected into each hemisphere at a rate of 4 nL/sec. The injection was delivered in 30 cycles, with 10 nL injected per cycle, followed by an 8-second pause between cycles to allow for virus diffusion in brain tissue. Following the final injection cycle, the micropipette remained *in situ* for 10 minutes to facilitate further viral diffusion before being slowly withdrawn. For optic fiber implantation, a ceramic sleeve (Precision Fiber Products, Inc.) was placed onto the ferrule of an optic fiber (CravitySci). The fiber assembly was then secured to a micromanipulator and lowered through the existing craniotomy to a depth of 2.3 mm below the dural surface. The brain surface surrounding the implanted fiber within the craniotomy was protected with a layer of vaseline or ophthalmic ointment to prevent contact with subsequently applied adhesives. A thin layer of dental cement (Parkell C & B Metabond) was applied over the protective ointment and the surrounding skull to create a dry base. A cone-shaped headcap was constructed around the exposed portion of the optic fiber ferrule using Loctite Gel Control cyanoacrylate adhesive to provide robust support. The adhesive was allowed to cure for two minutes. The micromanipulator was then detached, leaving the optic fiber and sleeve assembly implanted. This implantation procedure was replicated for the contralateral hemisphere at the symmetrical coordinates. A ceramic cap was inserted into each sleeve to prevent dust accumulation and maintain optical clarity for subsequent recordings. Additional cyanoacrylate adhesive gel was applied around both ferrules to reinforce the structural integrity of the implants, followed by a final protective layer of dental cement over the entire headcap assembly. The same procedure was repeated for both hemispheres.

### 4.7 Electrophysiology

Neural activity was recorded from DMS of fully trained mice using Neuropixels 2.0 multi-shank probes. Each probe has four 10mm shanks, with a total of 5120 recording sites across them. The acquisition system used included a National Instruments PXIe-1071 chassis, with a PXIe-8381 module. Neural signals were recorded with a sampling rate of 30 kHz across 384 channels using SpikeGLX software. Before recording, activity across the probe shanks was observed. The surface of the brain was located and used to estimate the band of recording sites most likely to cover the DMS, which the 384 channels were then assigned from which to record. Surface row information was saved to later validate histologically determined unit locations.

To do so, we stereotaxically implanted Neuropixel probes in fully trained mice using custom 3D printed fixtures for chronic, freely moving recordings, verifying the locations of all identified units *post hoc* (Figure 2b). After recovery from surgical probe implantation, mice were retrained and recorded from for up to a month, depending on stability. Recordings were preprocessed using built-in methods from SpikeInterface (Buccino et al., 2020) and sorted using a consensus-based algorithm combining Kilosort 2.5 and Kilosort 3 (Pachitariu et al., 2016), all of which were orchestrated and managed with the DataJoint infrastructure (Yatsenko et al., 2021).

### 4.8 Histology

At the end of the experiment, mice were anesthetized with isoflurane and injected with sodium pentobarbital (200mg/kg). Mice were then transcardially perfused with phosphate-buffered saline (PBS), then 4% paraformaldehyde (PFA). Brains were removed and placed in 4% PFA overnight, then transferred to PBS. Brains were sliced coronally at 50 μm using a vibratome (Leica VT1000 S), then mounted on glass slides. Prior to surgery, the probes were coated in Vybrant DiI (V22885) and the tracks were then imaged using a fluorescence microscope (Keyence BZ-X800). Probe track locations were reconstructed within the Allen Mouse Brain Atlas using HERBS (https://github.com/Whitlock-Group/HERBS).

### 4.9 Neural Data Preprocessing and Spike Sorting

Recording sessions were processed using a cloud-based pipeline implemented with DataJoint and running on AWS cloud services. Code for the preprocessing and sorting steps is publicly available at https://github.com/dj-sciops/jhu_shuler-lab_element-array-ephys.

Preprocessing was conducted using the SpikeInterface toolbox. First, a bandpass filter was applied to the raw signals to remove frequencies below 300 Hz and above 6000 Hz. Broken channels were identified and interpolated over so they would not disrupt the spike sorting algorithm. Phase shift correction was applied to account for the phase delays between channels during the system’s acquisition cycles. Lastly, common average referencing (CAR) was applied across all channels to reduce common noise artifacts.

Spike sorting was then performed with Kilosort 2.5 and Kilosort 3. These two sorting algorithms typically produced different sets of identified units, with a large portion of the units in both appearing to in truth be noise. The quality units identified consistently appeared in both algorithms, so the SpikeInterface consensus method was used to find the agreement between the two sorters and carry their intersection to the following manual curation step. Quality metrics were calculated for all consensus units, then assessed during manual curation performed using Phy, where units identified as noise were removed. The set of high quality units for each session was then finalized in the cloud pipeline, where they then became available for analysis.

### 4.10 Analysis - Behaviorally Projected Exit Time Calculation

To predict how long a given mouse on a given session would be willing to wait following each reward, we fit their leave times from the final rewards relative to the time of those rewards from nose-poke entry using simple linear regression.


LeaveTimeFromReward∼RewardTimefromPortEntry

Separate models were fit to trials from each context block, to allow for shifts in both the slope and intercept of the correlation. We then used the regression models to predict how long following every reward the mouse would have been willing to wait, if no further rewards had been delivered on that trial.

### 4.11 Analysis - Neural Step-like Classification

To characterize neurons exhibiting step-like responses at varying temporal delays from reward, an algorithm was developed to identify high and low firing rate states across trials and precisely detect the timing of transitions between states. To do so, first the instantaneous firing rate of individual neurons was calculated as one over the interspike interval for 10 ms time bins. For each unit, all of the post-reward responses in the time-investment port were pooled together and a sigmoid curve was fitted to the unsmoothed firing rate data. The parameters (y-offset, height, slope, and center) of this session sigmoid were then used as initial parameters for sigmoid fits to every interval individually. Parameter bounds were set to force the individual interval (reward-to-reward and reward-to-exit) sigmoid fits to match the high and low firing-rate states identified in the session sigmoid fit. The absolute value of the sigmoid slope was required to be greater than 11 (in units of delta(spikes/second)/second), preventing the sigmoid from fitting ramping activity with very shallow curves. The center parameter (inflection point of the sigmoid) was bounded to the duration of the interval plus or minus 10%. The primary criteria for a successful fit was for the sigmoid to fit the center within the interval (at least 100 ms after the start and 100 ms before the end). On intervals where there was no clear step, the sigmoid’s best fit pushed the center to one of the far edges of the interval, essentially fitting the interval as entirely low firing or entirely high firing, due to the strict bounds on the other parameters. As intervals could be shorter than the mean time to transition of any given unit, units were classified as step-like if the algorithm successfully identified a step on at least 50% of the intervals that were longer than the mean transition time plus one standard deviation.

### 4.12 Analysis - Neural Accumulation

Sessions with at least six step-like units were evaluated for the accumulation model of population activity. The cumulative number of units in their post-transition state was measured over the course of each trial. The slope and intercept of the accumulation across each reward-reward and reward-exit interval was calculated using simple linear regression. To predict the leave time from the neural activity, a cumulative unit threshold was first calculated based on the average number of transitioned units at the time of each true exit. Finally, the intersection of each interval regression line with the threshold was found, where the intersection time was the predicted leave time.

### 4.13 Analysis - Dopamine Data Processing and Transient Quantification

Fluorescence data were acquired using a commercial fiber photometry system (Neurophotometrics) at a sampling rate of 40 Hz. Two channels were used: a dopamine-dependent signal excited by a 470 nm LED and a dopamine-independent isosbestic signal excited by a 405 nm LED, which served as a motion control. Raw signals from both channels were first detrended using a Butterworth high-pass filter and then denoised with a 200 ms moving average filter.

To correct for motion artifacts, the 405 nm isosbestic signal was scaled to fit the 470 nm signal using linear regression. This fitted isosbestic signal was then subtracted from the 470 nm signal to produce a motion-corrected fluorescence signal.

Finally, the corrected signal was converted to the normalized change in fluorescence (ΔF/F0). This was calculated for each time point, t, using a moving baseline, where ΔF is the corrected increase in fluorescence signal, and F0 is the mean of the 470-nm signal in a 10-second window centered on that time point.

Quantification of a reward-evoked dopamine transient was achieved by peak amplitude computation. For each reward event, the peak was defined as the maximum ΔF/F0 value occurring within a 0.5-second window after the reward. The baseline was calculated as the minimum ΔF/F0 value within a 0.5-second window after the reward but before the peak. The peak amplitude was then determined by subtracting this baseline value from the peak value.

### 4.14 Analysis - Linear Mixed-Effects Model

To quantify how dopamine response to each reward is associated with reward time from entry, context block reward rate, and reward time from prior rewards, a linear mixed-effects model was performed using the following formula:

Dopaminepeakamplitude∼log(NRI)+RewardRateContext+log(IRI)

NRI denotes the entry (N)-to-reward (R) interval, and IRI denotes inter-reward interval. log(NRI) and log(IRI) were standardized prior to modeling.

## Supplementary Material

1
