# Supplementary material for: Reward-reset interval timing drives patch foraging decisions through neural state transitions in dorsomedial striatum
Source: bioRxiv. 2025 Oct 1:2025.09.29.679309. Preprint. [Version 1] doi: 10.1101/2025.09.29.679309 (PMC12621824; doi:10.1101/2025.09.29.679309)
Supplement: 1 [file NIHPP2025.09.29.679309v1-supplement-1.pdf]

bioRxiv preprint doi: https://doi.org/10.1101/2025.09.29.679309; this version posted October 1, 2025. The copyright holder for this preprint (which was not certified by peer review) is the author/funder, who has granted bioRxiv a license to display the preprint in perpetuity. It is made available under aCC-BY 4.0 International license.

5. Supplement

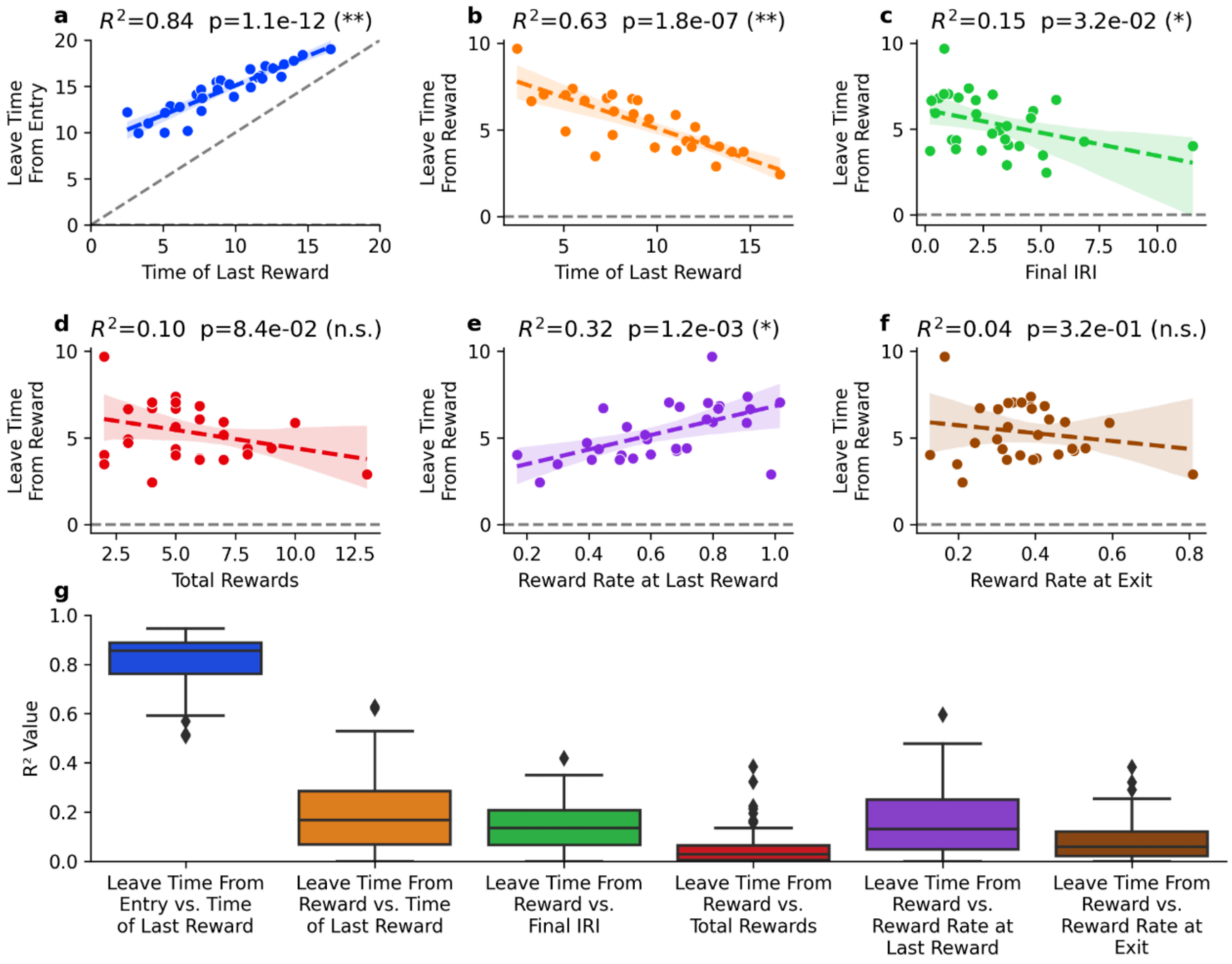

Supplemental Figure 1: Session-specific behavioral correlates of patch exit timing. a-f) Linear regression analysis of potential predictors of patch exit behavior from an example session. **a)** Leave time from port entry plotted against the time of the last reward relative to entry. **b)** Leave time from the final reward plotted against the time of the last reward relative to entry. **c)** Leave time from the final reward plotted against the duration of the final inter-reward interval. **d)** Leave time from the final reward plotted against the total number of rewards received in the time-investment port during that trial. **e)** Leave time from the final reward plotted against the reward rate experienced in the time between port entry and the final reward. **f)** Leave time from the final reward plotted against the reward rate experienced in the time between patch entry and patch exit. **g)** Distribution of correlation strengths (R^2) across all recording sessions for each of the six behavioral predictors analyzed in panels **a-f**. Each data point represents the R^2 value from the linear regression for a single session. Box plots show median, quartiles, and range of correlation strengths.

bioRxiv preprint doi: https://doi.org/10.1101/2025.09.29.679309; this version posted October 1, 2025. The copyright holder for this preprint (which was not certified by peer review) is the author/funder, who has granted bioRxiv a license to display the preprint in perpetuity. It is made available under aCC-BY 4.0 International license.

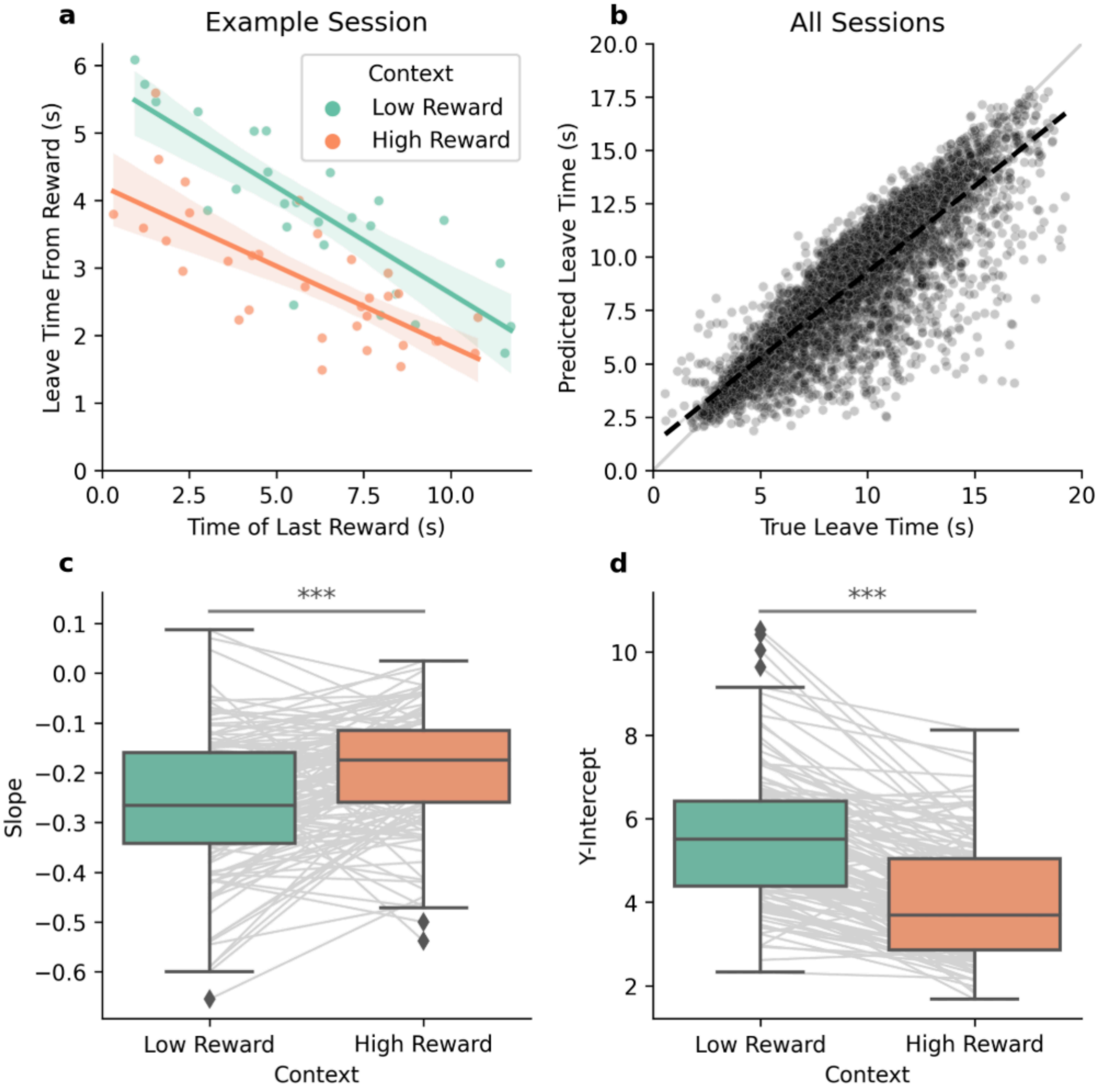

Supplemental Figure 2: Session-specific linear regression models predict behavioral exit policy following rewards. a) Leave time from the final reward plotted against the time of the final reward relative to port entry for an example session. Data points are separated by context block reward rate with fitted linear regression lines. **b)** Predicted leave times from port entry calculated using session-specific linear regression coefficients plotted against observed leave times across all sessions ($R^2 = 0.73$). **c)** Comparison of regression line slopes between high and low reward-rate context blocks across sessions ($n = 264$ sessions, $p = 2.5\times10^{-7}$, paired t-test). **d)** Comparison of regression line intercepts between high and low reward-rate context blocks across sessions ($n = 264$ sessions, $p = 1.8\times10^{-22}$, paired t-test).

bioRxiv preprint doi: https://doi.org/10.1101/2025.09.29.679309; this version posted October 1, 2025. The copyright holder for this preprint (which was not certified by peer review) is the author/funder, who has granted bioRxiv a license to display the preprint in perpetuity. It is made available under aCC-BY 4.0 International license.

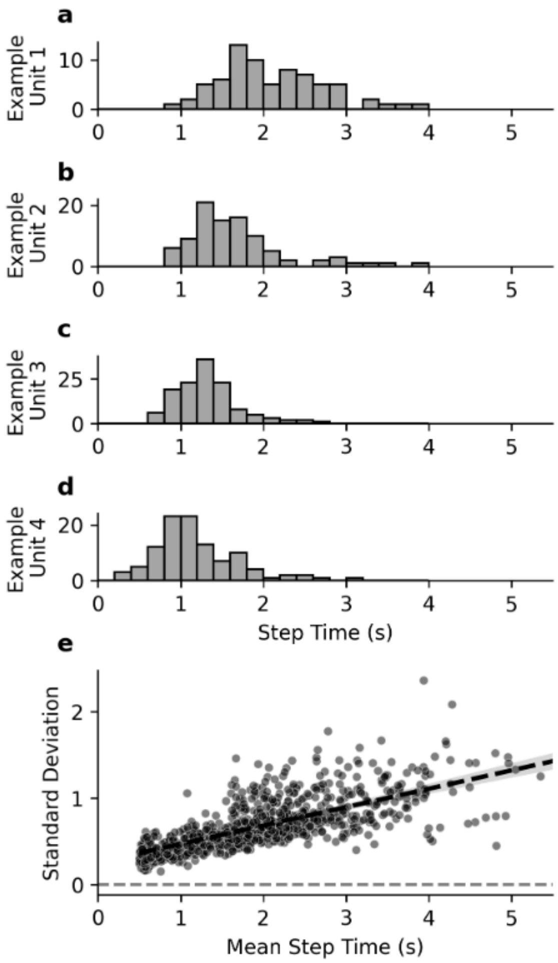

Supplemental Figure 3: Step time distributions and variability scaling in DMS neurons. a-d) Histograms showing the distribution of step times for the four example DMS units from Figures 2 and 3. **e)** Relationship between the mean step time and standard deviation of step times across all step-like units (n = 849 units). Linear regression line is shown in black ($R^2 = 0.318$, $p < 0.05$).

bioRxiv preprint doi: https://doi.org/10.1101/2025.09.29.679309; this version posted October 1, 2025. The copyright holder for this preprint (which was not certified by peer review) is the author/funder, who has granted bioRxiv a license to display the preprint in perpetuity. It is made available under aCC-BY 4.0 International license.

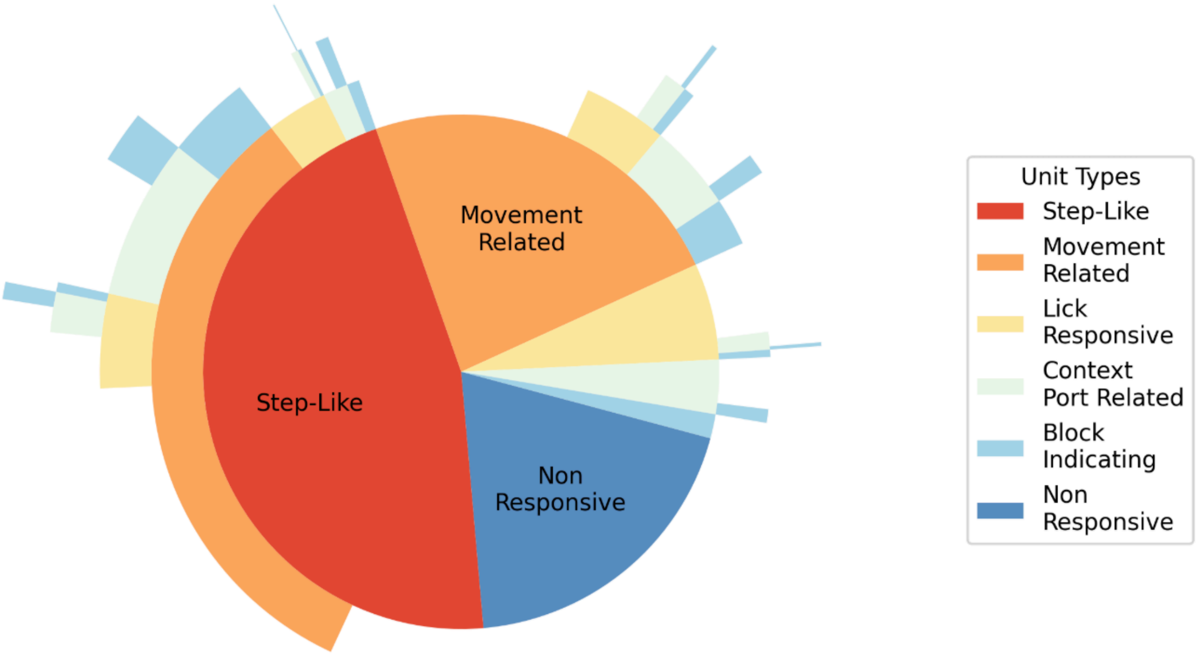

Supplemental Figure 4: Classification and overlap of DMS unit response types during patch-foraging behavior. Sunburst diagram showing the distribution of response types among all recorded DMS units (n = 1,845). Step-like units exhibit detectable step transitions in firing rate during the post-reward period in the time-investment port. Movement-related units show modulated activity during locomotion into or out of either port, which could be for general travel or port-specific entry/exit responses. Lick-responsive units display firing modulation phase-locked to the lick cycle during continuous licking behavior throughout port occupancy. Context port-related units exhibit activity modulation during the fixed inter-reward intervals in the context port or responses to the depletion cue. Block-indicating units modulate their firing rate during the initial 1.25 seconds following context port entry, before reward delivery, based on the expected reward-rate block from the previous trial. Non-responsive units show no detectable modulation to any measured task variables.

bioRxiv preprint doi: https://doi.org/10.1101/2025.09.29.679309; this version posted October 1, 2025. The copyright holder for this preprint (which was not certified by peer review) is the author/funder, who has granted bioRxiv a license to display the preprint in perpetuity. It is made available under aCC-BY 4.0 International license.

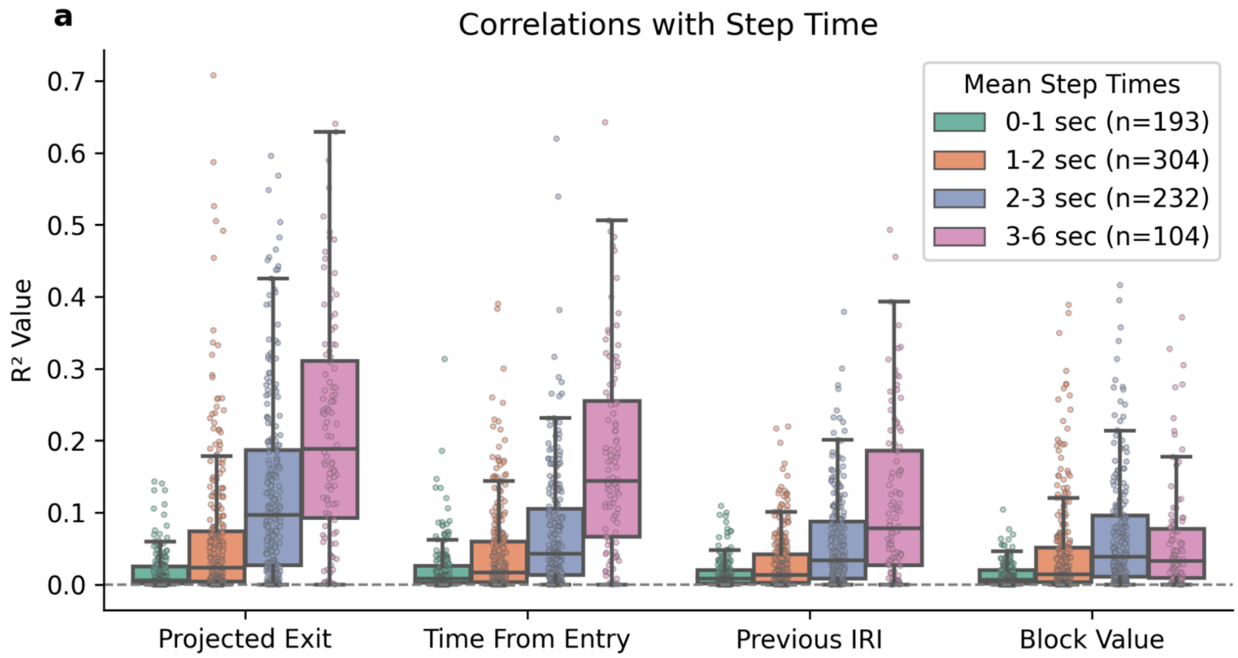

Supplemental Figure 5: Correlation strength between neural step times and task variables varies by mean step time of DMS units. Box plots showing the distribution of R^2 values quantifying the relationship between individual unit step times and four task variables: projected exit time (time the mouse would wait following each reward based on session-specific behavioral models), time of reward relative to port entry, previous inter-reward interval, and context block reward rate. Units are grouped by their mean step time. Individual R^2 values for each unit are overlaid as points using a swarm plot distribution to avoid overlap.
